# Training in extreme hot and cold

**DOI:** 10.1186/2052-1847-7-S1-O16

**Published:** 2015-08-11

**Authors:** Mike Tipton

**Affiliations:** 1 Extreme Environments Laboratory, DSES, University of Portsmouth, Portsmouth, PO1 2HP, UK

## 

Since the 5^th^ century BC consideration has been given to diet and training for improved physical performance. In contrast the impact of environmental temperature and humidity on performance has largely been ignored until recent times, despite the fact that the environmental threat can result in severe impairment of performance and even death.

As physical performance is impaired in both hot and cold environments, the challenge in these environments is to try, through various interventions, to maintain as closely as possible, elite performance. The thermal threats include: thermal discomfort detrimentally influencing concentration, dehydration, heat syncope, heat exhaustion, heat stroke, cold-induced neuromuscular dysfunction, cold injury, drowning and hypothermia.

Strategies and interventions to reduce the impact of environmental extremes on performance include: selection strategies, clothing design, acclimation and acclimatisation protocols, fluid replacement strategies, artificial sweating, active cooling and chemical or psychological perceptual adjustment.

Which if these interventions are appropriate depends on the mode of exercise to be undertaken, the specific nature of the thermal threat and the characteristics of the athlete in question. It follows that for optimal performance maintenance at the elite level, these considerations should be conducted on an individual basis, and include a detailed an analysis of the conditions to be faced and thermoregulatory characteristics of the athlete in question.

## References

[B1] Tipton MJ Golden F Immersion in cold water: effects on performance and safety Chapter in: Oxford Textbook of Sports Medicine 1998 2nd Edition Oxford University Press, Oxford

[B2] Tipton MJ Environmental Factors. Chapter in: ABC of Sports Medicine 2005 Oxford University Press

[B3] Tipton Mj Pandolf K Sawka M Werner J Talor NAS Nigel A.S. Taylor, Herbert Groeller and Peter L. McLennan Physiological adaptation to hot and cold environments. Chapter in: Human performance in the cold: the physiology of acute cold exposure Physiological bases of human performance during work and exercise 2007

